# Targeting ataxia telangiectasia-mutated- and Rad3-related kinase (ATR) in PTEN-deficient breast cancers for personalized therapy

**DOI:** 10.1007/s10549-018-4683-4

**Published:** 2018-02-02

**Authors:** Nouf Al-Subhi, Reem Ali, Tarek Abdel-Fatah, Paul M. Moseley, Stephen Y. T. Chan, Andrew R. Green, Ian O. Ellis, Emad A. Rakha, Srinivasan Madhusudan

**Affiliations:** 1 Translational Oncology, Division of Cancer and Stem Cells, School of Medicine, University of Nottingham, Nottingham University Hospitals, Nottingham, NG5 1PB UK; 2 Department of Oncology, Nottingham University Hospitals, Nottingham, NG5 1PB UK; 3 Department of Pathology, Division of Cancer and Stem Cells, School of Medicine, University of Nottingham, Nottingham University Hospitals, Nottingham, NG5 1PB UK

**Keywords:** Breast cancer, Biomarker, PTEN, ATR, Triple-negative breast cancer, Synthetic lethality

## Abstract

**Purpose:**

Phosphate and tensin homolog (PTEN), a negative regulator of PI3K signaling, is involved in DNA repair. ATR is a key sensor of DNA damage and replication stress. We evaluated whether ATR signaling has clinical significance and could be targeted by synthetic lethality in PTEN-deficient triple-negative breast cancer (TNBC).

**Methods:**

PTEN, ATR and pCHK1^Ser345^ protein level was evaluated in 1650 human breast cancers. ATR blockade by VE-821 was investigated in PTEN-proficient- (MDA-MB-231) and PTEN-deficient (BT-549, MDA-MB-468) TNBC cell lines. Functional studies included DNA repair expression profiling, MTS cell-proliferation assay, FACS (cell cycle progression & γH2AX accumulation) and FITC-annexin V flow cytometry analysis.

**Results:**

Low nuclear PTEN was associated with higher grade, pleomorphism, de-differentiation, higher mitotic index, larger tumour size, ER negativity, and shorter survival (*p* values < 0.05). In tumours with low nuclear PTEN, high ATR and/or high pCHK1^ser345^ level was also linked to higher grade, larger tumour size and poor survival (all *p* values < 0.05). VE-821 was selectively toxic in PTEN-deficient TNBC cells and resulted in accumulation of double-strand DNA breaks, cell cycle arrest, and increased apoptosis.

**Conclusion:**

ATR signalling adversely impact survival in PTEN-deficient breast cancers. ATR inhibition is synthetically lethal in PTEN-deficient TNBC cells.

**Electronic supplementary material:**

The online version of this article (10.1007/s10549-018-4683-4) contains supplementary material, which is available to authorized users.

## Introduction

Phosphate and tensin homolog (PTEN) is a key tumor suppressor [5, 7, 14, 22]. Mutations in PTEN have been reported in several tumours including brain, prostrate, melanoma, endometrial and breast cancers. Cytoplasmic PTEN serves as a negative regulator of PI3K/AKT signaling pathway. More recently, nuclear PTEN has been shown to have roles in DNA repair [5, 7, 14, 22]. Ataxia telangiectasia-mutated- and Rad3-related protein (ATR), a serine threonine kinase belonging to the PIKK family (phosphoinositide 3-kinase-like-family of protein kinase), is a critical regulator of DNA repair and genomic integrity [9, 10, 19, 20]. ATR can be activated by single stranded (ss)–double stranded (ds) DNA junctions generated at sites of DNA damage, during nucleotide excision repair, at resected double-strand breaks and stalled replication forks. Activated ATR in turn phosphorylates Chk1 at Ser^345^ and Ser^317^, as well as several other target proteins involved in homologous recombination repair and DNA cross-link repair. Phosphorylation of Chk1 at Ser^345^ (pChk1) leads to its activation which further coordinates cell cycle progression and DNA repair [9, 10, 19, 20].

In the current study, we provide evidence that ATR and pChk1 expression in of PTEN-deficient breast cancer adversely impact on survival. ATR inhibition is synthetically lethal in PTEN-deficient TNBC cells implying a promising personalized therapy approach in breast cancers.

## Patients and methods

### Clinical study

#### Patient characteristics and tissue microarray (TMA)

The Nottingham Tenovus Primary Series is a well-characterized consecutive cohort of early-stage primary operable invasive BC patients from which the samples were used for the TMA construction [1, 2, 4, 21]. Patient demographics are summarized in Supplementary Table S1. Breast cancer specific survival (BCSS) was defined as the number of months from diagnosis to the occurrence of BC-related death. Survival was censored if the patient was still alive at the time of analysis, lost to follow-up, or died from other causes.

#### Immunohistochemistry (IHC)

A mouse monoclonal PTEN antibody clone 6H2.1 (Dako, Denmark) was diluted in 1:20 dilution using Leica Antibody diluent. Antigen retrieval was performed as follows: the TMA slides were incubated at 98 °C (in water bath) for 25 min in target retrieval solution (pH 9.0; Leica Microsystems, Newcastle, UK) then the slides were incubated for 10 min at room temperature in warm Tris buffer saline (TBS, pH 7.6, 50 °C). Finally, slides were cooled by flooding in cold water (pH 6.0) for 5 min. The primary antibody was incubated for 1 h at room temperature. The Novocastra Novolink Max Polymer Detection Systems (RE7280-K: 1250 tests, Leica Biosystems) was used to visualise the staining. Briefly, the slides were heated at 60 °C for 10 min for dewaxing. Following cooling of samples to room temperature, the slides were rehydrated using a Leica Autostainer. After performing antigen retrieval, the slides were loaded onto Shandon Cover plates and fitted on sequenza plates for IHC. Initially, Endogenous peroxidase was blocked for 5 min, and then a protein block was applied for 5 min. The PTEN antibody was added, and the slides were incubated for 1 h at room temperature. After this, post-primary block was run for 30 min and then polymer was applied for further 30 min. The chromogen (DAB) was applied for 5 min, and tissues were counter-stained with haematoxylin for 6 min. Slides were dehydrated using Leica Autostainer and mounted with DPX. Negative (omission of the primary antibody) and positive controls were included according to manufacturer datasheet. ATR and pChk1 staining have been reported previously. Optimisation, specificity and cut-off points of ATR and pChk1 antibodies used in the current study have been reported previously [1]. A set of slides were incubated for 18 h at 4 °C with the primary mouse monoclonal anti-ATR antibody, clone 1E9 (H00000545-M03, Novus Biologicals, Cambridge, UK), at a dilution of 1:20. A further set of slides were incubated for 60 min with the primary rabbit polyclonal anti-phosphorylated Chk1 antibody (Ab58567, Abcam, Cambridge, UK), at a dilution of 1:140 [1].

#### Evaluation of immunohistochemical staining

The TMA slides were initially assessed through staining quality and specificity under light microscope. Slides were then scanned into high-resolution digital images (0.45 lm/pixel) using a NanoZoomer slide scanner (Hamamtsu Photonics, Welwyn Garden City, UK) and accessed using a web-based interface (Distiller, SlidePath Ltd, Dublin, Ireland). They were scored at ×20 magnification using a minimum of 2400 high-resolution screen (1920 × 1080). Assessment of staining was based on a semi-quantitative approach using a modified histochemical score (*H*-score) taking the intensity of staining and the percentage of stained cells into account. For the intensity, a score index of 0, 1, 2, and 3 was used, which corresponded to negative, weak, moderate, and strong staining intensity, respectively. The percentage of positive cells per intensity was estimated. PTEN staining in breast tumour cells was detected in the nuclei and cytoplasm of the breast cancer cells. Cut-off was used to classify the tumours with a nuclear-PTEN *H*-score equal to ‘0’ as negative, and those with an *H*-score more than 0 as positive. *H*-score > 90 was taken as the cut-off for high cytoplasmic-PTEN expression. Optimisation, specificity and cut-off points of ATR and pChk1 antibodies used in the current study have been reported previously [1]. H-score of ≥ 60 was taken as the cut-off for high ATR expression, H-score of ≥ 50 was taken as the cut-off for high cytoplasmic pChk1 expression and *H*-score of ≥ 50 was taken as the cut-off for high nuclear pChk1 expression.

Tumor marker prognostic studies (REMARK) criteria, recommended by McShane et al. [13], were followed throughout this study. Ethical approval was obtained from the Nottingham Research Ethics Committee (C202313).

#### Statistical analysis

Statistical analysis was performed using SPSS v 22 statistical software. Association with clinical and biological markers was assessed using Chi-squared test. Kaplan–Meier survival curve with log-rank test was plotted to determine the survival distribution of studied patients’ subgroups. Statistical significance was defined as *p* value ≤ 0.05. Due to multiple comparisons, the adjustment for *p* values of multiple testing was used according to Benjamini–Hochberg correction method.

### Pre-clinical study

#### Cell lines, tissue culture and chemical reagents

MDA-MB-231 human breast cancer cell line was purchased from American Type culture collection (ATCC, Manassas, USA) and was grown as per ATCC recommendations. MDA-MB-231 cells were cultured in RPMI-1640 medium (Sigma, St. Louis, MO, USA) and Minimum Essential Medium Eagle (Sigma-Aldrich) supplemented with 1% l-glutamine (200 mM) and 1% non-essential amino acids (0.1 mM), respectively. The breast cancer cell lines BT-549 and MDA-MB-468 (Cell Line Service, Eppelheim, Germany) were cultured in Dulbecco’s Modified Eagle Medium/Nutrient Mixture F-12 (Gibco, Carlsbad, USA). All media were supplemented with 10% foetal bovine serum (Sigma) and 1% penicillin/streptomycin (10,000 units penicillin and 10 mg streptomycin/mL) (Sigma, Gillingham, UK). All cell lines were cultured as adherent cultures in a humidified 5% CO_2_ incubator at 37 °C. ATR inhibitor (VE-821, 10 mM in 1 mL DMSO) was purchased from Selleckchem (Houston, TX, USA). DMSO (Sigma, Gillingham, UK) was used as solvent to dissolve VE-821 and was tested solely as a vehicle control (< 0.1% v/v).

#### qRT-PCR analysis of DNA repair gene expression in breast cancer cell lines

Real-time PCR was performed using RT^2^ Profiler DNA Repair PCR Array for 84 DNA repair genes in duplicates as described previously [3]. The data were analyzed as per manufacturer’s recommendations. GAPDH was used for normalization of the data. A twofold change or above in expression was considered significant. All experiments were performed in duplicate.

#### Western blot analysis

To evaluate the specificity of PTEN-antibody used for the immunohistochemical study, cell lysates were prepared and Western blot analysis was performed. The primary antibodies for PTEN, ATR, and β-actin used in this study were incubated at room temperature for 1 h (*PTEN* 1:100 dilution [Dako], *ATR* 1:1000 dilution [Cell signaling] and *β*-*actin* 1:5000 dilution [Sigma]). Infrared dye-labelled secondary antibodies (Li-Cor) [IRDye 800CW Donkey Anti-Rabbit IgG and IRDye 680CW Donkey Anti-Mouse IgG] were incubated at a dilution of 1:10,000 for 1 h. Membranes were scanned with a Li-Cor Odyssey machine (700 and 800 nm) to determine protein expression.

#### MTS cell proliferation assay

Cells were seeded at a density of 1000 cells per well in 96-well plates and allowed to adhere overnight. Inhibitory compounds were added to samples in plates at a range of concentrations after 16 h, followed by incubation of plates for total of 5 days. All steps of MTS assay were performed as per manufacturer’s recommendations. All experiments were performed in triplicate at least three times. Data analysis was performed in Microsoft Excel 2010 (Microsoft, Redmond, USA) and GraphPad Prism 6 (GraphPad, La Jolla, USA).

#### γH2AX accumulation and cell cycle analysis by flow cytometry

1 × 10^5^ cells were seeded in 6-well tissue culture plates, and were allowed to adhere overnight. VE-821, an ATR inhibitor, was added after 16 h at the concentration of 5 μM. Cells were harvested by trypsinisation and centrifuged at 1000 rpm for 5 min at 24 and 48 h post drug exposure. Cells pellets were then re-suspended in 1 mL of 70% ice-cold ethanol to fix the cells. Following this, samples were stored at 4 °C. Suspensions were centrifuged at 1000 rpm for 5 min followed by removal of the supernatant. Samples were processed using H2AX Phosphorylation Assay Kit (Merckmillipore, Nottingham, UK) as per manufacturer’s recommendations. At least 10,000 cells from each sample were analysed. All experiments were done in duplicate three times. Weasel (Victoria, Australia) flow cytometry analysis software was used for data analysis. Graphical representation and statistical analysis was performed in GraphPad Prism 6 (GraphPad, La Jolla, USA).

#### Apoptosis detection by FITC-Annexin V flow cytometry

1 × 10^5^ cells were seeded into 6-well tissue culture plates and allowed to adhere overnight. VE-821 was added to tissue culture plates at a concentration of 5 μM after 16 h. 24 and 48 h after VE-821 administration. For detection of apoptosis, FITC-Annexin V flow cytometry was performed using the fluorescein isothiocyanate [FITC]-Annexin V Apoptosis Detection Kit I (BD Pharmingen, San Jose, USA) as per manufacturer’s recommendations. At least 10,000 cells from each sample were analysed. Weasel (Victoria, Australia) flow cytometry analysis software was used for data analysis. The percentage of apoptotic cells (FITC-Annexin V positive, PI positive and FITC-Annexin V positive, PI negative) in the treated population was ascertained by comparing it to a control population of untreated samples. Statistical analysis was performed in GraphPad Prism 6 (GraphPad, La Jolla, USA). All experiments were done in duplicate three times.

## Results

### Loss of nuclear PTEN and aggressive breast cancers

**Fig. 1:**
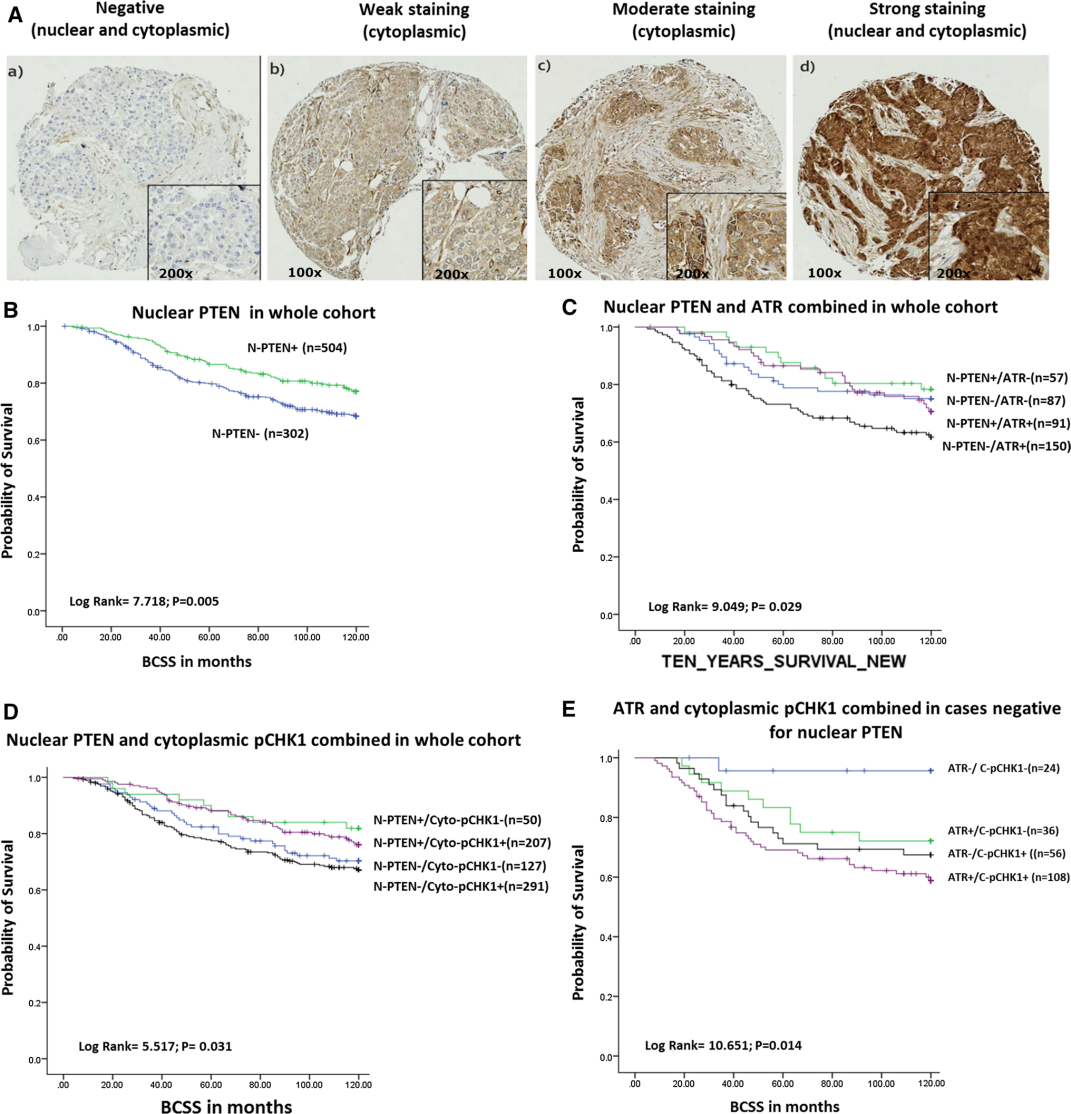
**a** Nuclear and cytoplasmic staining of PTEN [a negative staining for both, b negative staining for nuclei and weak for cytoplasm, c some weak staining for nuclei and moderate for cytoplasm d strong staining for both; TMA cores pictures were taken using digital pathology interference at ×100 (left) and ×200 (right)]. Kaplan–Meier plot showing breast cancer-specific survival (BCSS) and **b** nuclear PTEN level. **c** combined nuclear PTEN and ATR level. **d** combined nuclear PTEN and cytoplasmic pCHK1 level. **e** combined ATR and cytoplasmic pCHK1 level nuclear PTEN negative tumours

The loss of nuclear PTEN expression was observed in 508/811 (62.6%) tumours compared with 303/811 (37.4%) tumours which had positive expression of nuclear (PTEN). Low cytoplasmic PTEN expression was seen in 326/811 (40.2%) tumours compared with the 485/811 (59.8%) tumours which had high cytoplasmic expression (Fig. 1a). The negative nuclear PTEN was associated with higher grade, less tubule formation, pleomorphism, higher mitotic index, larger tumour size, high-risk Nottingham Prognostic Index (NPI), ER− and PR− tumours (all adjusted *p* values < 0.01) (Table 1). Similarly, low cytoplasmic PTEN was also associated with aggressive phenotype including high grade, de-differentiation, higher mitotic index and larger tumours (all adjusted *p* values < 0.005) (Table 2).

**Table 1:** Association between nuclear-PTEN expression and clinicopathological variables

Parameters	Negative (%)	Positive (%)	*p* value	Adjusted *p* value
Grade			**2.2** **×** **10**^**−7**^	**1.6** **×** **10**^**−6**^
1	49 (9.7)	55 (18.3)		
2	149 (29.6)	122 (40.5)		
3	305 (60.6)	124 (41.2)		
Tubules			**0.001553**	**0.0021742**
1	20 (4.1)	17 (5.8)		
2	144 (29.3)	118 (40.4)		
3	328 (66.7)	157 (53.8)		
Pleomorphism			**0.000667**	**0.00116725**
1	8 (1.6)	5 (1.7)		
2	154 (31.4)	131 (44.9)		
3	329 (67.0)	156 (53.4)		
Mitosis			**0.000001**	**0.0000035**
1	123 (25.0)	121 (41.4)		
2	94 (19.1)	59 (20.2)		
3	275 (55.9)	112 (38.4)		
Stage			0.475148	3.3250
I	294 (58.4)	189 (62.8)		
II	167 (33.2)	89 (29.6)		
III	42 (8.3)	23 (7.6)		
Tumour size			**0.020606**	**0.024040333**
< 2.0	218 (43.3)	156 (51.7)		
≥ 2.0	286 (56.7)	146 (48.3)		
NPI			**0.000012**	**0.000028**
> 0.3	107 (22.3)	109 (37.8)		
3.4–5.4	291 (60.6)	147 (51.0)		
> 5.4	82 (17.1)	32 (11.1)		
ER status			**8.6** **×** **10**^**−11**^	**2.5** **×** **10**^**−10**^
Negative	175 (34.8)	42 (13.9)		
Positive	328 (65.2)	261 (86.1)		
PR status			**8** **×** **10**^**−11**^	**4.8** **×** **10**^**−10**^
Negative	251 (51.6)	82 (27.9)		
Positive	235 (48.4)	212 (72.1)		
HER2 status			0.512577	0.6150924
Negative	412 (84.6)	241 (82.8)		
Positive	75 (15.4)	50 (17.2)		

Bold = statistically significant result

**Table 2:** Association between cytoplasmic-PTEN expression and clinicopathological variables

Parameters	Low (%)	High (%)	*P* value	Adjusted *p* value
Grade			**0.002740**	**0.006393333**
1	27 (8.3)	77 (16.0)		
2	107 (33.0)	164 (34.2)		
3	190 (58.6)	239 (49.8)		
Tubules			**0.000169**	**0.001183**
1	9 (2.9)	28 (6.0)		
2	84 (26.7)	178 (38.0)		
3	222 (70.5)	263 (56.1)		
Pleomorphism			0.991045	6.937315
1	5 (1.6)	8 (1.7)		
2	114 (36.3)	171 (36.5)		
3	195 (62.1)	290 (61.8)		
Mitosis			**0.000455**	**0.0015925**
1	85 (27.0)	159 (33.9)		
2	48 (15.2)	105 (22.4)		
3	182 (57.8)	205 (43.7)		
Stage			0.466340	0.544063333
I	203 (62.7)	280 (58.3)		
II	96 (29.6)	160 (33.3)		
III	25 (7.7)	40 (8.3)		
Tumour size			**0.012480**	**0.017472**
< 2.0	133 (41.0)	241 (50.0)		
≥ 2.0	191 (59.0)	241 (50.0)		
NPI			**0.003111**	**0.00544425**
> 0.3	69 (22.4)	147 (32.0)		
3.4–5.4	198 (64.3)	240 (52.2)		
> 5.4	41 (13.3)	73 (15.9)		
ER status			**0.000004**	**0.000012**
Negative	115 (35.7)	102 (21.1)		
Positive	207 (64.3)	382 (78.9)		
PR status			**0.000319**	**0.0004785**
Negative	158 (50.5)	175 (37.5)		
Positive	155 (49.5)	292 (62.5)		
HER2 status			**0.000008**	**0.000016**
Negative	286 (91.1)	367 (79.1)		
Positive	28 (8.9)	97 (20.9)		

Bold = statistically significant result

### PTEN, ATR, and pChk1^ser345^ co-expression and survival

A previous preclinical study has shown that loss of PTEN and activation of AKT can impair Chk1 through phosphorylation, reduce nuclear localization, promote cytoplasmic sequestration, ubiquitination and degradation [18]. Loss of nuclear PTEN can also promote genomic instability and DNA strand breaks which can activate ATR. We, therefore, investigated PTEN, ATR, and pChk1^ser345^ co-expression in clinical breast cancers. A strong association between negative nuclear PTEN and negative nuclear pChk1 expression was observed. Interestingly, there was also a significant association between low cytoplasmic PTEN and low ATR as well low-cytoplasmic Chk1 expression (Table 3**)**. We then proceeded to evaluate prognostic significance of PTEN, ATR, and pChk1^ser345^ co-expression.

**Table 3:** Association between PTEN, ATR and pChk1expression in breast cancers

Biomarkers	PTEN (nuclear) negative (%)	PTEN (nuclear) positive (%)	Unadjusted *p* value	Adjusted *p* value
ATR			0.736306	4.417836
Negative	87 (36.6)	57 (38.3)		
Positive	151 (63.4)	92 (61.7)		
Nuclear pChk1			**5.3** **×** **10**^**−8**^	**1.1** **×** **10**^**−7**^
Negative	294 (63.5)	122 (43.1)		
Positive	169 (36.5)	161 (56.9)		
Cytoplasmic pChk1			**0.002627**	**0.0039405**
Negative	127 (30.2)	51 (19.8)		
Positive	293 (69.8)	207 (80.2)		

Biomarkers	PTEN (cytoplasmic) low (%)	PTEN (cytoplasmic) high (%)	Unadjusted *p* value	Adjusted *p* value
ATR			**0.005322**	**0.0063864**
Negative	66 (46.2)	78 (32.0)		
Positive	77 (53.8)	166 (68.9)		
Nuclear pChk1			0.396997	2.381982
Negative	169 (57.7)	247 (54.5)		
Positive	124 (42.3)	206 (45.5)		
Cytoplasmic pChk1			**8.2** **×** **10**^**−7**^	**4.9** **×** **10**^**−6**^
Negative	96 (36.8)	82 (19.7)		
Positive	165 (63.2)	335 (80.3)		

Bold = statistically significant result

The nuclear PTEN negativity was associated with shorter breast cancer-specific survival (BCSS) (*p* = 0.005) compared to nuclear PTEN-positive breast cancer (Fig. 1b). In contrast cytoplasmic PTEN did not influence survival (Supplementary Fig. S1A). When combined together, we observed that tumours with nuclear PTEN-negative/cytoplasmic PTEN-positive tumours had the worst BCSS (*p* = 0.039) (Supplementary Fig. S1B).

We then investigated the prognostic significance of PTEN, ATR and pChk1^ser345^ co-expression in patients. Nuclear PTEN negative/high ATR tumours were associated with the worst BCSS (*p* = 0.029) (Fig. 1c). Similarly, nuclear PTEN-negative/high-cytoplasmic pCHK1^ser345^ tumours were associated with poor survival (*p* = 0.031) (Fig. 1d). Nuclear PTEN-negative/high-nuclear pCHK1^ser345^ tumours were also associated with poor survival (*p* = 0.037) (Supplementary Fig. 1c). In nuclear PTEN-negative tumours, we then investigated the prognositic significance of ATR- cytoplasmic pCHK1^ser345^ co-expression. As shown in Fig. 1e, tumours with high-ATR and -cytoplasmic pCHK1^ser345^ expression had the worst survival compared to those with low-ATR and-cytoplasmic expression (*p* = 0.014).

ATR and/or pCHK1^ser345^ did not influence survival in tumours with low- or high-cytoplasmic PTEN expression (Supplementary Fig. S1D–S1G).

Taken together, the clinical data suggest that nuclear PTEN deficiency as well as ATR and pChk1^ser345^ expression in nuclear PTEN-deficient breast cancers is associated with aggressive breast cancers.

### PTEN-deficient breast cancer cell lines demonstrate deregulation in gene expression of multiple DNA repair pathways

**Fig. 2:**
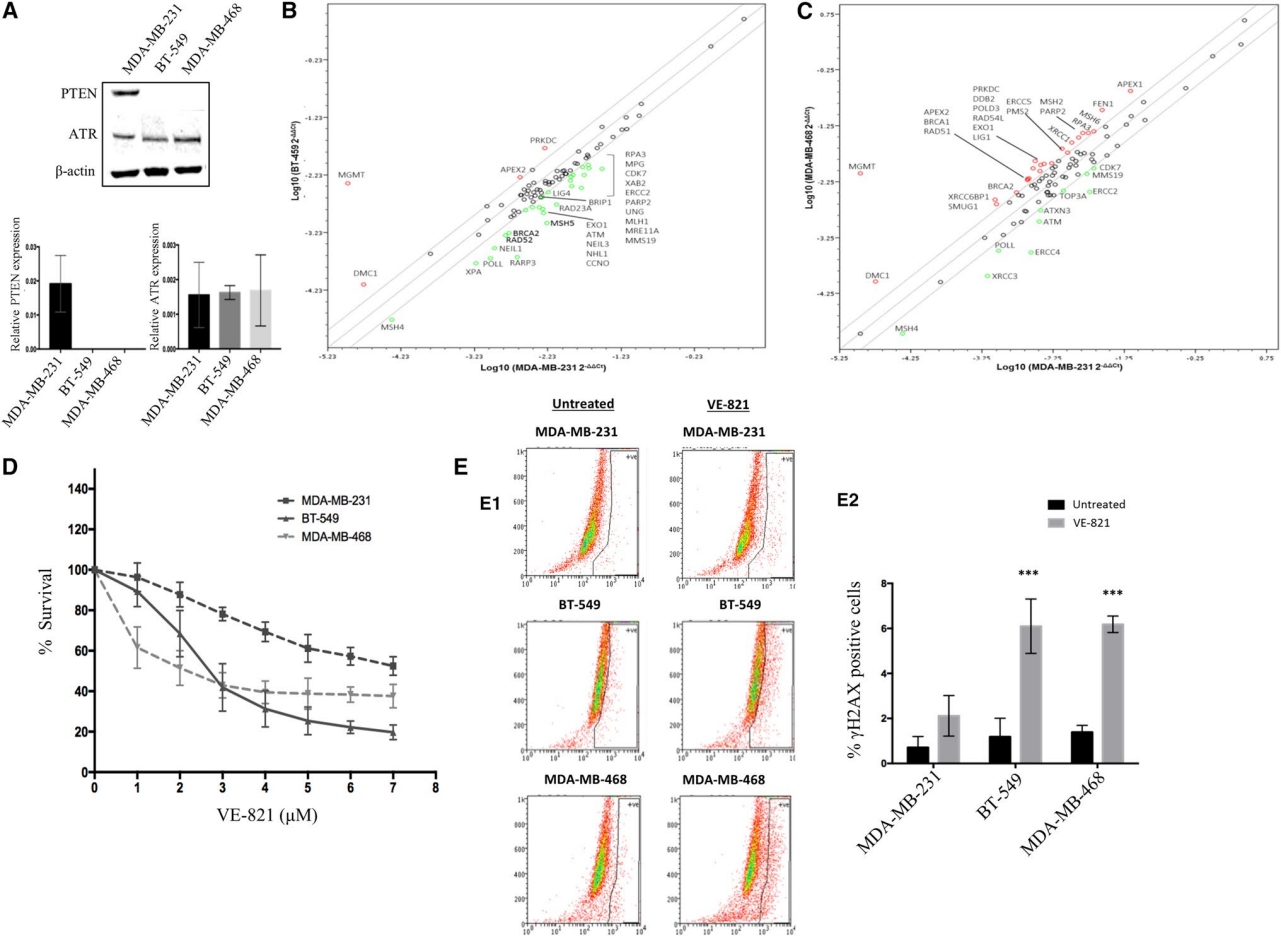
**a** Western blotting analysis for PTEN, ATR expression in breast cancer cell lines. **b** DNA repair expression profiling in BT549 cells compared to MDA-MB-231 cells. **c** DNA repair expression profiling in MDA-MB-468 cells compared to MDA-MB-231 cells. **d** MTS growth inhibition assay in a panel of PTEN-proficient and—deficient human BC cells treated with VE-821. **e** γH2AX accumulation by FACS in BC cells treated with VE821

We initially screened a panel of TNBC cell lines for expression of PTEN, ATR, total Chk1 and pChk1^ser345^. As shown in Fig. 2a, MDA-MB-231 cell line is PTEN-proficient, whereas BT-549 and MDA-MB-468 cell lines are PTEN deficient. We did not observe any significant differences in the expression of ATR in MDA-MB-231, BT-549 and MDA-MB-468 TNBC cell lines (Fig. 2a). Previous studies suggest that nuclear PTEN may have roles in DNA repair including in homologous recombination repair. To test whether BT-549 and MDA-MB-468 cells have altered DNA repair expression compared to MDA-MB-231 cells, we profiled the mRNA level expression of 84 DNA repair genes using the RT^2^-Profiler DNA Repair PCR array. The expression of DNA repair genes in BT-549 and MDA-MB-468 cells was compared with DNA repair expression in MDA-MB-231. The data are shown in Fig. 2b, c as well as in Supplementary Tables S2–S7. We observed more than two-fold downregulation of several genes in HR, BER, NHEJ, MMR and NER. Interestingly, in MDA-MB-468, we observed upregulation of PARP2, XRCC6BP1, LIG1, RPA3 and MGMT. In BT-549 cells, DMC1, XRCC6BP1 and MGMT were significantly upregulated. The data would not only concur with previously published data but also support the hypothesis that PTEN deficiency can alter DNA repair expression status in cells.

### VE-821 (ATR inhibitor) is selectively toxic in PTEN deficient TNBC cells

Given the current pharmaceutical interest in the development of ATR inhibitors for cancer therapy, we pre-clinically tested whether ATR inhibition could be selectively toxic in PTEN-deficient cells. VE-821 is a highly selective ATR kinase inhibitor with an IC_50_ of 26 nM in cell-free assays for ATR inhibition. In MTS cell proliferation assays, we observed that VE-821 was more toxic to BT-549 and MDA-MB-468 compared to MDA-MB-231 cells (Fig. 2d).

**Fig. 3:**
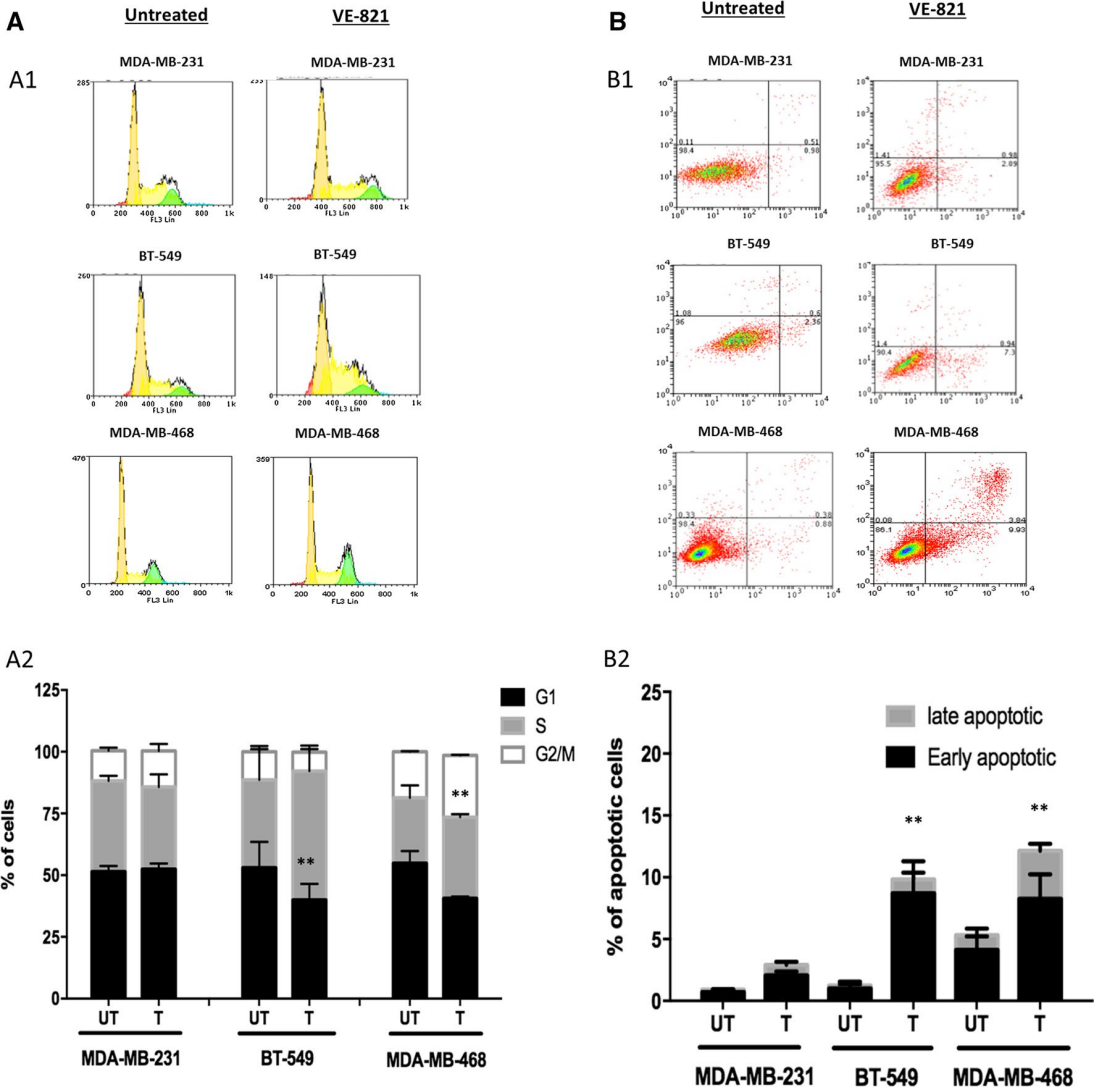
**a** Cell cycle analysis by FACS in BC cells treated with VE821. **b** Apoptosis detection by Annexin V-FITC FACS in BC cells treated with VE821

Phosphorylated H2AX (γH2AX) is a marker of double-strand breaks. As expected, there was substantial γH2AX accumulation [thereby suggesting double-strand break (DSBs) accumulation] in BT-549 and MDA-MB-468 cells compared to MDA-MB-231 cells after 24 h of VE-821 treatment (Fig. 2E1, E2). The accumulation of DSBs was associated with S-phase arrest in BT-549 cells and G2/M arrest in MDA-MB-468 cells after 48 h of VE-821 treatment (Figs. 3A1, 3A2). Cell cycle arrest was subsequently followed by accumulation of apoptotic cells (Figs. 3B1, 2B2).

Taken together, the data provides evidence that VE-821 induces synthetic lethality in PTEN-deficient cells through accumulation of DSBs, cell cycle arrest and induction of apoptosis.

## Discussion

We provide the first clinical evidence that ATR signaling could have adverse impact on survival in PTEN-deficient breast cancers. In addition, the pre-clinical evidence presented here also suggests that ATR inhibition could be a promising synthetic lethality strategy in PTEN-deficient breast cancers.

Loss of not only cytoplasmic PTEN expression but also nuclear PTEN expression was associated with aggressive ER−, PR− and HER2− phenotypes. Our data concurs with previous studies that correlate PTEN loss with TNBCs [6, 8, 16]. In addition, the negative PTEN expression status either in cytoplasm or nucleus resulted in negative expression of nuclear or cytoplasmic pCHK1^ser345^. The data are consistent with a previous study showing that the loss of PTEN-impaired Chk1 through phosphorylation mediated by elevated AKT and subsequent Chk1 ubiquitination [18]. Puc et al. also demonstrated that PTEN loss reduces Chk1 nuclear localization, thereby increasing the genomic instability and promoting tumorigenesis [18]. Supporting these observations, our study demonstrated that cytoplasmic/nuclear PTEN loss resulted in significantly increasing tumour size, mitotic index and NPI.

Whereas cytoplasmic PTEN serves as a negative regulator of PI3K/AKT signaling, nuclear PTEN has been shown to have roles in DNA repair [5, 7, 14, 22]. For example, PTEN loss has been shown to trigger downregulation of both RAD51 [15, 23] and BRCA1 [17]. Accordingly, we observed that DNA repair profiling in PTEN-deficient cells, altered expression of several genes involved multiple DNA repair pathways including BER, NHEJ, HR, NER and MMR. The data suggest that PTEN may be directly or indirectly involved in the regulation of multiple DNA repair genes.

Pharmaceutical development of ATR inhibitors for cancer therapy is an attractive anti-cancer strategy [9, 10]. Pre-clinically, we observed that PTEN deficient breast cancer cells were sensitive to ATR inhibition and was associated with accumulation of DSBs, cell cycle arrest and induction of apoptosis. The data imply that PTEN-deficient cells with impaired DNA repair may be reliant upon ATR signaling pathway for survival. ATR blockade in this context could lead to synthetic lethality. Recently, ATM inhibition was also shown to be synthetically lethal in PTEN-deficient cells [12]. Interestingly, ATM-deficient cells are also sensitive to ATR inhibitors via synthetic lethality in leukemic cells [11].

In conclusion, we have demonstrated that ATR signaling adversely impact upon survival in PTEN-deficient breast cancers. ATR targeting could be a promising synthetic lethality approach in PTEN-deficient breast cancers.

## Electronic supplementary material

Below is the link to the electronic supplementary material.

Supplementary material 1 (DOCX 13 kb)

Supplementary material 2 (TIFF 479 kb)

Supplementary material 3 (TIFF 118 kb)

Supplementary material 4 (DOCX 36 kb)
