# Supplementary material for: Targeting ataxia telangiectasia-mutated- and Rad3-related kinase (ATR) in PTEN-deficient breast cancers for personalized therapy
Source: Breast Cancer Res Treat. 2018 Feb 2;169(2):277–86. doi: 10.1007/s10549-018-4683-4 (PMC5945733; doi:10.1007/s10549-018-4683-4)
Supplement: Supplementary file 1 — Supplementary material 1 (DOCX 13 kb) [file 10549_2018_4683_MOESM1_ESM.docx]

**Supplementary Figure S1.** Kaplan Meier plot showing breast cancer specific survival (BCSS) and **A.** cytoplasmic PTEN level in whole series. **B.** combined cytoplasmic PTEN and ATR level in whole series. **C.** combined cytoplasmic PTEN and cytoplasmic pCHK1 level in whole series **D.** combined cytoplasmic PTEN and cytoplasmic pCHK1 level in whole series **F.** combined ATR and cytoplasmic pCHK1 level in patients who were negative for cytoplasmic PTEN.
