# Supplementary figures and images for: Targeting ataxia telangiectasia-mutated- and Rad3-related kinase (ATR) in PTEN-deficient breast cancers for personalized therapy
Source: Breast Cancer Res Treat. 2018 Feb 2;169(2):277–86. doi: 10.1007/s10549-018-4683-4 (PMC5945733; doi:10.1007/s10549-018-4683-4)

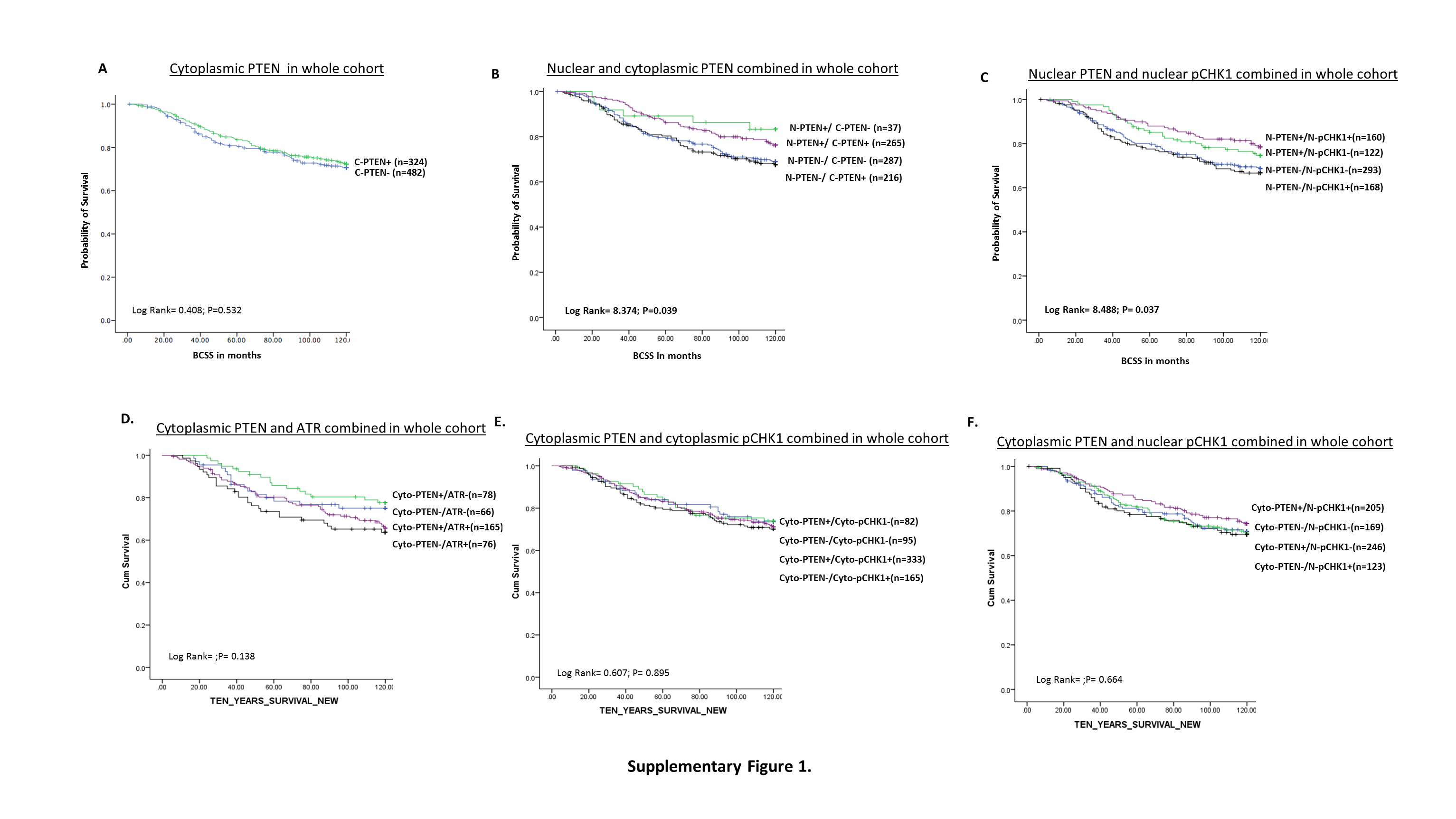

Supplement: Supplementary file 2 — Supplementary material 2 (TIFF 479 kb) [file 10549_2018_4683_MOESM2_ESM.tif]

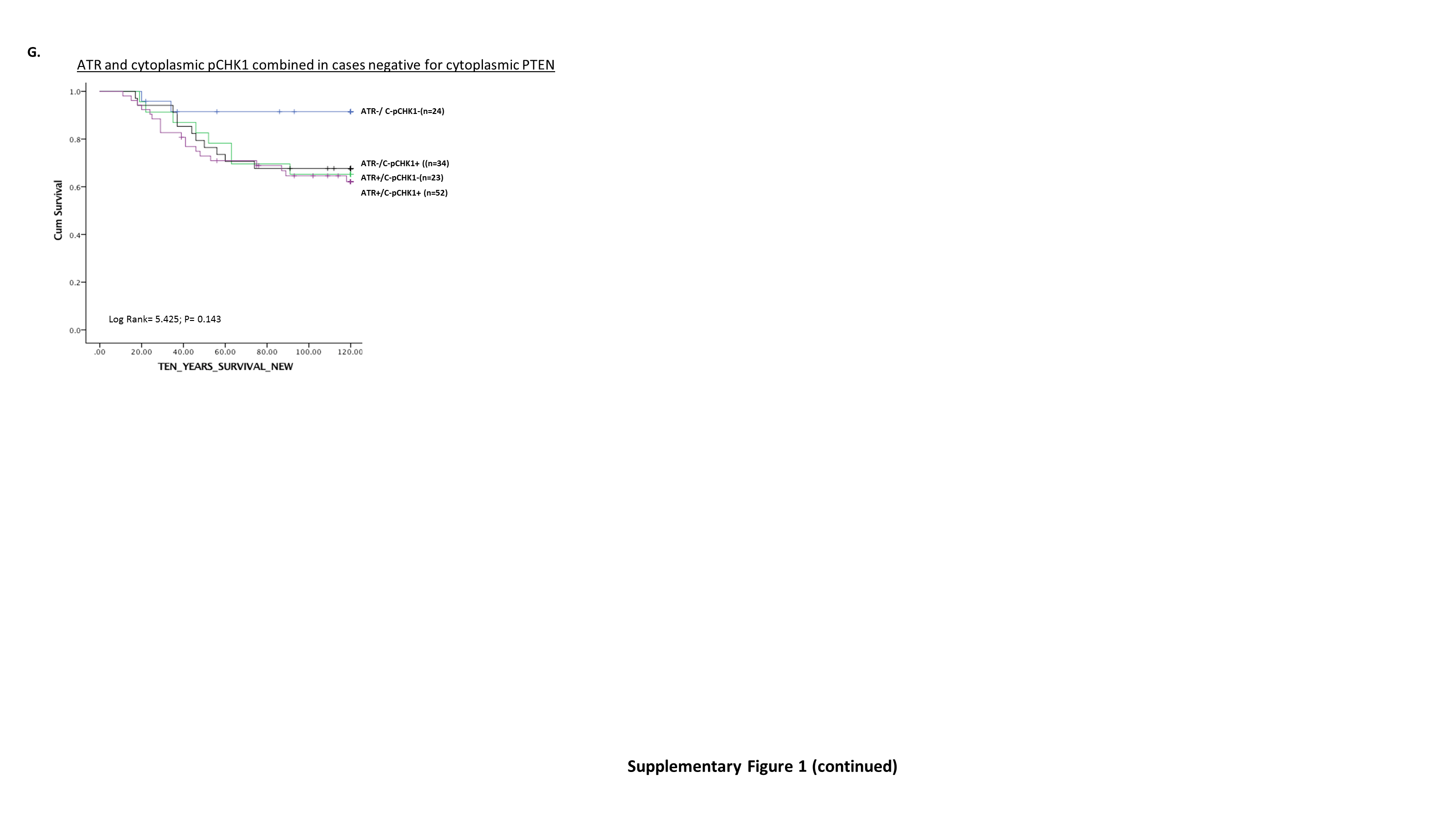

Supplement: Supplementary file 3 — Supplementary material 3 (TIFF 118 kb) [file 10549_2018_4683_MOESM3_ESM.tif]
