# Supplementary material for: Targeting ataxia telangiectasia-mutated- and Rad3-related kinase (ATR) in PTEN-deficient breast cancers for personalized therapy
Source: Breast Cancer Res Treat. 2018 Feb 2;169(2):277–86. doi: 10.1007/s10549-018-4683-4 (PMC5945733; doi:10.1007/s10549-018-4683-4)
Supplement: Supplementary file 4 — Supplementary material 4 (DOCX 36 kb) [file 10549_2018_4683_MOESM4_ESM.docx]

**Supplementary Table S1:** Clinicopathological characteristics of Nottingham Tenovus series.

| **Variable** | **Cases (%)** |
| --- | --- |
| **Menopausal status** |  |
| Pre-menopausal | 612 (37.0) |
| postmenopausal | 1038 (63.0) |
| **Tumour Grade (NGS)** |  |
| G1 | 306 (18.5) |
| G2 | 531 (32.2) |
| G3 | 813 (49.3) |
| **Lymph node stage** |  |
| Negative | 1056 (64.0) |
| Positive (1-3 nodes) | 486 (29.5) |
| Positive (>3 nodes) | 108 (6.5) |
| **Tumour size (cm)** |  |
| T1 a + b (≤1.0) | 187 (11.0) |
| T1 c (>1.0 -2.0) | 868 (53.0) |
| T2 (>2.0-5) | 579 (35.0) |
| T3 (>5) | 16 (1.0) |
| **Tumour type** |  |
| IDC-NST | 941 (57) |
| Tubular | 349 (21) |
| ILC | 160 (10) |
| Medullary (typical/atypical) | 41 (2.5) |
| Others | 159 (9.5) |
| **NPI subgroups** |  |
| Excellent PG(2.08-2.40) | 207 (12.5) |
| Good PG(2.42-3.40) | 331 (20.1) |
| Moderate I PG(3.42 to 4.4) | 488 (29.6) |
| Moderate II PG(4.42 to 5.4) | 395 (23.9) |
| Poor PG(5.42 to 6.4) | 170 (10.3) |
| Very poor PG(6.5–6.8) | 59 (3.6) |
| **Survival at 20 years** |  |
| Alive and well | 1055 (64.0) |
| Dead from disease | 468 (28.4) |
| Dead from other causes | 127 (7.6) |
| **Adjuvant systemic therapy (AT**) |  |
| No AT | 665 (42.0) |
| Hormone therapy (HT) | 642 (41.0) |
| Chemotherapy | 307 (20.0) |
| Hormone + chemotherapy | 46 (3.0) |

* Number of cases for which data were available.

NPI; Nottingham prognostic index, PG; prognostic group

**Supplementary Table S2.** BER genes with altered gene expression in PTEN deficient BT-549 and MDA-MB-468 cells compared to PTEN proficient cells. P-values were determined using two-tailed unpaired student t-test.

| Base Excision Repair Pathway | BT-549 versus MDA-MB-231 | | MDA-MB-468 versus MDA-MB-231 | |
| --- | --- | --- | --- | --- |
|  | Fold Change | P value | Fold change | P value |
| APE1 | 0.8409 | 0.644 | 3.41 | 0.177 |
| APE2 | 2.1409 | 0.136 | 2.56 | 0.315 |
| PARP1 | 0.6646 | 0.306 | 1.231 | 0.645 |
| PARP2 | 0.4513 | 0.069 | 2.691 | 0.057 |
| PARP3 | 0.0974 | 0.070 | 0.753 | 0.460 |
| Pol β | 0.8289 | 0.188 | 1.765 | 0.073 |
| SMUG1 | 1.0783 | 0.945 | 2.50 | 0.300 |
| NTHL1 | 0.2969 | 0.037 | 1.122 | 0.631 |
| UNG | 0.4508 | 0.338 | 1.544 | 0.544 |
| FEN1 | 0.6177 | 0.468 | 3.95 | 0.152 |
| OGG1 | 1.9623 | 0.148 | 1.592 | 0.496 |
| NEIL1 | 0.2803 | 0.213 | 0.555 | 0.379 |
| NEIL2 | 0.6761 | 0.108 | 0.704 | 0.081 |
| NEIL3 | 0.3544 | 0.103 | 1.594 | 0.300 |
| XRCC1 | 0.5896 | 0.142 | 2.694 | 0.067 |
| CCNO | 0.2496 | 0.120 | 0.628 | 0.353 |
| LIG3 | 0.5788 | 0.082 | 0.793 | 0.851 |
| MUTYH | 0.7783 | 0.592 | 1.70 | 0.578 |

**Supplementary Table S3.** HR pathway genes with altered gene expression in PTEN deficient BT-549 cells compared to PTEN proficient cells. P-values were determined using two-tailed unpaired student t-test.

| Homologous Recombination pathway Genes | BT versus 231 | | 468 versus 231 | |
| --- | --- | --- | --- | --- |
|  | Fold Change | P value | Fold change | P value |
| BRCA1 | 1.0125 | 0.973063 | 2.532 | 0.156862 |
| BRCA2 | 0.332 | 0.20239 | 2.058 | 0.230322 |
| RAD21 | 0.9083 | 0.658759 | 0.652 | 0.267711 |
| RAD50 | 0.654 | 0.283137 | 1.735 | 0.120646 |
| RAD51B | 0.6843 | 0.337469 | 0.523 | 0.273475 |
| RAD51C | 0.5739 | 0.160754 | 1.536 | 0.202792 |
| RAD51D | 1.2799 | 0.939635 | 1.729 | 0.587591 |
| RAD54L | 0.9831 | 0.770987 | 4.103 | 0.213757 |
| RAD52 | 0.3349 | 0.273047 | 0.636 | 0.482307 |
| XRCC2 | 0.5976 | 0.495719 | 1.744 | 0.70808 |
| XRCC3 | 0.8049 | 0.706057 | 0.173 | 0.087876 |
| ATM | 0.4435 | 0.228304 | 0.297 | 0.190541 |
| DMC1 | 4.0699 | 0.056979 | 5.178 | 0.283165 |

**Supplementary Table S4.** NHEJ pathway genes with altered gene expression in PTEN deficient BT-549 and MDA-MB-468 cells compared to PTEN proficient cells. P-values were determined using two-tailed unpaired student t-test.

| Non-homologous end joining pathway Genes | | BT-549 versus MDA-MB-231 | | | MDA-MB-468 versus MDA-MB-231 | |
| --- | --- | --- | --- | --- | --- | --- |
|  | Fold Change | | P value | Fold change | | P value |
| DNA-PKcs | 3.1767 | | 0.151938 | 2.2424 | | 0.196427 |
| XRCC4 | 0.6943 | | 0.235329 | 0.7773 | | 0.294372 |
| XRCC5 | 0.6016 | | 0.190123 | 1.4348 | | 0.474652 |
| XRCC6 | 0.6525 | | 0.21293 | 1.7663 | | 0.151416 |
| XRCC6BP1 | 1.9332 | | **0.038247** | 3.1041 | | **0.009402** |
| LIG4 | 0.4924 | | 0.064275 | 1.3758 | | 0.500142 |

**Supplementary Table S5.** NER pathway genes with altered gene expression in PTEN deficient BT-549 and MDA-MB-468 cells compared to PTEN proficient cells. P-values were determined using two-tailed unpaired student t-test.

| Nucleotide Excision Repair Pathway | | BT-549 versus MDA-MB-231 | | | MDA-MB-468 versus MDA-MB-231 | |
| --- | --- | --- | --- | --- | --- | --- |
|  | Fold Change | | P value | Fold change | | P value |
| BRIP1 | 0.4956 | | 0.380722 | 0.9789 | | 0.94284 |
| LIG1 | 0.5405 | | 0.202439 | 2.3439 | | **0.038526** |
| ATXN3 | 0.7828 | | 0.517317 | 0.455 | | 0.341401 |
| RAD23A | 0.2282 | | **0.040881** | 0.8829 | | 0.863456 |
| RAD23B | 0.596 | | 0.17283 | 0.9852 | | 0.88487 |
| RPA1 | 0.7202 | | 0.594418 | 1.1208 | | 0.766113 |
| CCNH | 0.5783 | | 0.200337 | 0.7189 | | 0.364546 |
| CDK7 | 0.3626 | | **0.033358** | 0.4628 | | **0.0368** |
| DDB1 | 0.5131 | | 0.118977 | 1.1882 | | 0.576579 |
| DDB2 | 0.7791 | | 0.480425 | 2.758 | | 0.15099 |
| PNKP | 0.7202 | | 0.475932 | 1.1627 | | 0.818808 |
| POLL | 0.2203 | | 0.111151 | 0.3397 | | 0.105248 |
| RPA3 | 1.2153 | | **0.013077** | 2.4142 | | **0.011999** |
| SLK | 0.5859 | | 0.105294 | 0.5387 | | **0.011999** |
| ERCC1 | 0.6378 | | 0.449785 | 0.8553 | | 0.726503 |
| ERCC2 | 0.3126 | | 0.214585 | 0.2004 | | 0.182421 |
| ERCC3 | 0.6408 | | 0.441911 | 1.9336 | | 0.353102 |
| ERCC4 | 1.1797 | | 0.749765 | 0.1096 | | 0.12279 |
| ERCC5 | 0.7156 | | 0.155827 | 2.8671 | | 0.114258 |
| ERCC6 | 0.5076 | | 0.307206 | 0.7834 | | 0.680832 |
| ERCC8 | 0.5965 | | 0.237951 | 0.6749 | | 0.370102 |
| XAB2 | 0.2312 | | 0.077427 | 0.5188 | | 0.169362 |
| XPA | 0.2823 | | 0.053668 | 0.9 | | 0.925235 |
| XPC | 1.7057 | | 0.506012 | 0.6168 | | 0.279155 |

**Supplementary Table S6**. Genes related DNA repair with altered gene expression in PTEN deficient BT-549 and MDA-MB-468 cells compared to PTEN proficient cells. P-values were determined using two-tailed unpaired student t-test.

| Other related Genes | BT-549 versus MDA-MB- 231 | | MDA-MB-468 versus MDA_MB-231 | |
| --- | --- | --- | --- | --- |
|  | Fold Change | P value | Fold change | P value |
| ATR | 0.898 | 0.70747 | 1.6469 | 0.192378 |
| EXO1 | 0.4901 | 0.328215 | 3.3103 | 0.24269 |
| MGMT | 380.5219 | **3.87E-05** | 730.693 | **0.009658** |
| RAD18 | 0.7234 | 0.427313 | 1.0555 | 0.925664 |
| TOP3A | 0.8697 | 0.550096 | 0.4878 | 0.214917 |
| TOP3B | 0.638 | 0.367829 | 1.6348 | 0.378102 |
| MRE11A | 0.3016 | 0.178277 | 0.5998 | 0.3634 |
| MMS19 | 0.2073 | 0.181555 | 0.4552 | 0.287729 |
| TDG | 0.5845 | **0.016382** | 1.1143 | 0.491562 |

**Supplementary Table S7.** MMR related DNA repair with altered gene expression in PTEN deficient BT-549 and MDA-MB-468 cells compared to PTEN proficient cells. P-values were determined using two-tailed unpaired student t-test.

| Mismatch Repair pathway Genes | BT-549 versus MDA-MB-231 |  | MDA-MB-468 versus MDA_MB-231 |  |
| --- | --- | --- | --- | --- |
|  | Fold Change | P value | Fold change | P value |
| POLD3 | 1.1125 | 0.969898 | 2.9283 | 0.322472 |
| PMS1 | 0.7638 | 0.247354 | 1.3166 | 0.484463 |
| PMS2 | 0.6174 | 0.378696 | 2.0421 | 0.354854 |
| MLH1 | 0.3949 | 0.327065 | 0.9371 | 0.833309 |
| MLH3 | 0.564 | 0.352031 | 0.6934 | 0.595247 |
| MSH2 | 0.6145 | 0.396381 | 2.8051 | 0.142376 |
| MSH3 | 0.717 | 0.418346 | 0.8239 | 0.625614 |
| MSH4 | 0.413 | 0.398092 | 0.2465 | 0.254666 |
| MSH5 | 0.1484 | **0.030613** | 0.5616 | 0.29892 |
| MSH6 | 0.5834 | 0.303527 | 2.1445 | 0.304173 |
| MPG | 0.4148 | 0.277013 | 0.6059 | 0.406125 |
| TREX1 | 1.177 | 0.627562 | 1.304 | 0.575987 |
